# Idiopathic dendriform pulmonary ossification diagnosed only after a surgical biopsy: A case report

**DOI:** 10.1016/j.rmcr.2025.102227

**Published:** 2025-05-02

**Authors:** Tomohito Takeshige, Ryo Koyama, Eisuke Goto, Tatsuo Maeyashiki, Akifumi Okajima, Toshihiko Nishioki, Junko Watanabe, Toshifumi Yae, Takamitsu Banno, Kenji Kido, Kazuhisa Takahashi

**Affiliations:** a Department of Respiratory Medicine, Juntendo University Nerima Hospital, 3-1-1, Takanodai, Nerima-ku, Tokyo, 177-8521, Japan; b Department of Thoracic Surgery, Juntendo University Nerima Hospital, 3-1-1, Takanodai, Nerima-ku, Tokyo, 177-8521, Japan; c Department of Respiratory Medicine, Juntendo University Faculty of Medicine and Graduate School of Medicine, 3-1-3, Hongo, Bunkyo-ku, Tokyo, 113-8431, Japan

**Keywords:** Diffuse pulmonary ossification (DPO), Idiopathic dendriform pulmonary ossification (IDPO), Transbronchial lung biopsy (TBLB), Transbronchial lung cryobiopsy (TBLC)

## Abstract

Idiopathic dendriform pulmonary ossification (IDPO) presents as dendritic ossification of the lungs without a particular cause. IDPO is often diagnosed using surgical biopsy, transbronchial lung biopsy (TBLB), or transbronchial lung cryobiopsy (TBLC). In this case report, we described a 36-year-old male who presented with abnormal chest findings radiographically. TBLB was performed but no diagnosis was made. Instead, IDPO was diagnosed following a surgical biopsy. CT tomography and respiratory function tests showed no progression over the past 3.5 years. However, a nationwide survey in Japan revealed several cases of disease progression. Considering this, careful monitoring of the disease is recommended.

## Highlights

• IDPO presents as dendritic ossification of the lungs without a particular cause.
• In this case report, IDPO was diagnosed following a surgical biopsy.
• A nationwide survey in Japan revealed several cases of disease progression.
• Careful monitoring of the disease is recommended.

## 1 Introduction

Diffuse pulmonary ossification (DPO) is a rare disease characterized by diffuse heterotopic ossification within the lungs, which causes small, diffusely high-density granular shadows in the lung fields. It was first described by Luschka et al., in 1868. DPO is classified as idiopathic when the cause is unknown. It can also be classified by the underlying diseases. Morphologically, DPO is classified into two types: branching (dendriform) and nodular.

Idiopathic dendriform pulmonary ossification (IDPO) has a dendriform histological pattern. The prevalence of IDPO is approximately 0.17 in 100,000 individuals [1]. Diagnosis is often made surgically but can also be determined by TBLB or TBLC. In this case report, we described a patient with IDPO diagnosed after a surgical biopsy because a diagnosis was not possible with TBLB.

## 2 Case report

**Fig. 1:**
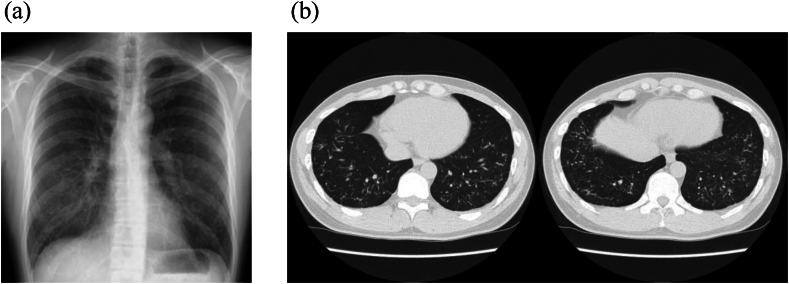
Image findings. (a) Diffuse granular shadows in both lung fields. (b)Bilateral branching, round, and linear structures.

**Fig. 2:**
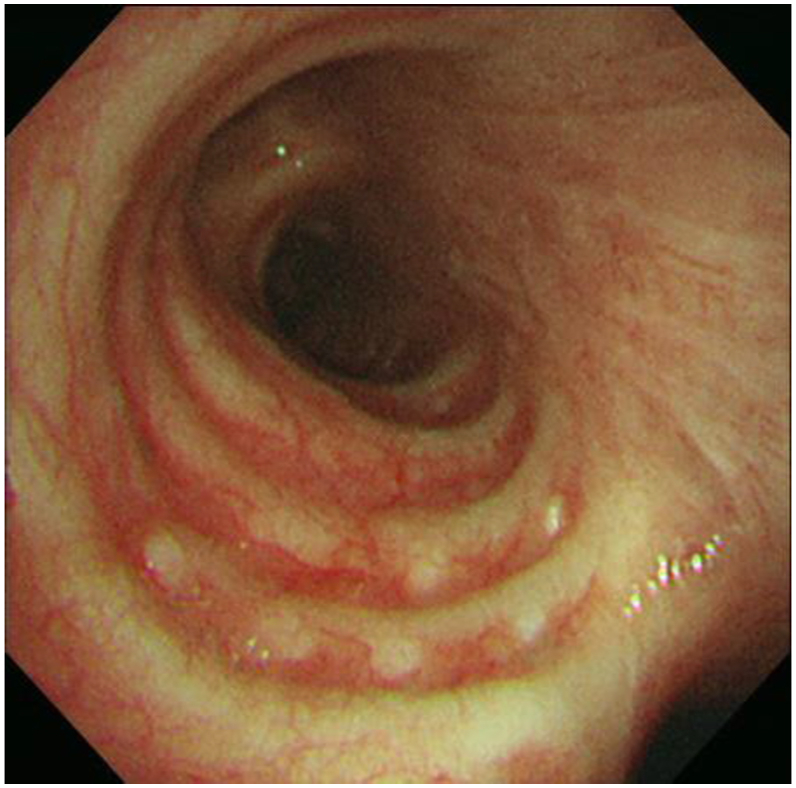
Bronchoscopy findings. In the right main bronchus, nodular mucosal lesions were found in a string of beads between the cartilage rings.

**Fig. 3:**
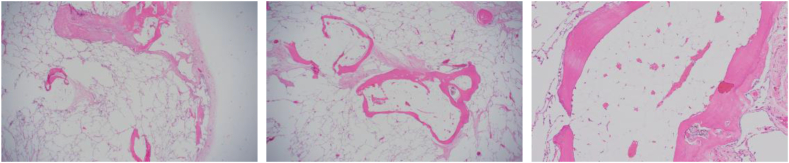
Histological findings. Diffuse large and small coral-like and trabecular bone formations in the lungs, with fatty marrow.

A 36-year-old male patient presented to our hospital with an abnormal shadowing on his chest radiograph, taken during a medical checkup. He had no notable medical or family history, history of smoking, or occupational history of dust or asbestos exposure. Auscultation did not reveal any significant findings. Chest radiography revealed bilateral diffuse granular shadows in the lung fields (Fig. 1a). Chest computed tomography (CT) revealed bilateral branching, round, and linear structures, with lower lobe predominance (Fig. 1b). Echocardiography revealed normal cardiac function. Respiratory function tests revealed a vital capacity of 4.70 L (95.9 % of predicted), FEV_1_/FVC of 76.4 %, and a carbon monoxide diffusing capacity of 21.7 mL/min/mmHg. The %DLCO was predicted to be 70.2 %. The results indicated reduced diffusibility of the lungs. Blood test results are presented in Table 1. Endocrine or electrolyte disorders, hypercalcemia, or hyperparathyroidism were not detected. Bronchoscopy was performed to investigate the cause of the abnormal chest shadowing. TBLB was performed at four sites in the right lung (Fig. 2), demonstrating benign alveolar tissue. Subsequently, a surgical biopsy, namely, a thoracoscopic partial right lower lobectomy, was performed. White convex pleural changes were observed in the visceral pleura of the right lower lobe. Multiple hard nodules were palpable in the lung specimens. Histological findings revealed diffuse large and small coral-like formations and trabecular bone formations in the lungs with fatty marrow (Fig. 3). No background disease was identified, and thus, the patient was diagnosed with IDPO. Throughout the three and a half years of follow-up, no particular changes were noted in the respiratory function tests or chest CT scans.

**Table 1:** Laboratory findings.

WBC	7000	/μL	Na	140	mM/L	PTH-intact	35	pg/mL
Neutrophils	71.9	%	K	4	mM/L	calcitonin	2.86	pg/mL
Lymphocytes	19.9	%	Cl	105	mM/L	PTHrP	<1.0	pmol/L
Eosinophils	1.9	%	Ca	9.2	mg/dL			
Monocytes	5.9	%	P	3.3	mg/dL			
RBC	503	× 104	Glu	88	mg/dL			
Hb	15	g/dL						
Hct	44.3	%	CRP	0.1	mg/dL			
Plt	26.2	× 104	IgG	1345	mg/dL			
			IgA	242	mg/dL			
TP	7.7	g/dL	IgM	107	mg/dL			
ALB	4.7	g/dL	IgE	40.3	IU/mL			
AST	19	IU/L	ACE	9.4	U/L			
ALT	20	IU/L	KL-6	230	U/mL			
ALP	172	IU/L	SP-D	23.5	ng/mL			
γ-GTP	37	IU/L	ANA	40	×			
LDH	170	IU/L	Rheumatoid factor	5	U/mL			
BUN	15	mg/dL	PR3-ANCA	<1.0	U/mL			
Cre	0.79	mg/dL	MPO-ANCA	<1.0	U/mL

## 3 Discussion

As mentioned, DPO can be classified into two types: idiopathic or associated with underlying diseases. In IDPO, the cause is not identified. Conversely, non-idiopathic types are broadly divided into those associated with lung, heart, and metabolic diseases. Histologically, DPO demonstrates ectopic bone formation. They are morphologically classified into two types: branching (dendriform) and nodular type [1]. In this case report, we described a patient for whom the underlying disease could not be identified and the pathology showed dendritic ossification. Consequently, the patient was diagnosed with IDPO.

The diagnosis of DPO is often made using a surgical biopsy. In some cases, the disease is diagnosed using TBLB [2,3]. However, there are also cases in which TBLB may not yield significant findings, and the diagnosis is made only after a surgical biopsy [[4], [5], [6], [7], [8], [9], [10]]. TBLC usually yields larger samples with less risks of crushing compared to TBLB. The literature also reports a case of DPO which was diagnosed by cryobiopsy, suggesting the potential relevance of crybiopsy for diagnosing DPO [11]. CT findings of branched, linear, and round structures within the lungs, with or without high attenuation, are indicative of IDPO [12]. Similar findings were observed on CT in our case patient. If CT findings are suggestive of IDPO, surgical biopsy or TBLC may be considered rather than TBLB.

In this case, bronchoscopy revealed a string of nodular mucosal lesions between the cartilage rings in the right main bronchus, suggesting tracheobronchopathia osteochondroplastica (TO). TO is a rare benign disorder affecting the airway cartilage and is characterized by abnormal nodular chondrification and ossification [13]. One case of TO and dendriform pulmonary ossification occurring simultaneously has been reported [14], suggesting that a common pathogenetic mechanism may exist.

In Japan, a nationwide survey of DPO found that the condition was more prevalent among young men (82 % male; mean age at diagnosis: 37.9 years). Based on CT findings, 88 % of patients experienced disease progression. Additionally, over 50 % with the condition experienced progressive deterioration of their respiratory function. A high KL-6 level has also been associated with disease progression [1]. In contrast, in our patient, significant changes in respiratory function and on CT imaging were not detected during the three and a half years of follow-up. Given the possibility of disease progression, careful continual observation of the patient's progress is necessary.

## CRediT authorship contribution statement

**Tomohito Takeshige:** Writing – original draft, Conceptualization. **Ryo Koyama:** Writing – review & editing. **Eisuke Goto:** Writing – review & editing. **Tatsuo Maeyashiki:** Writing – review & editing. **Akifumi Okajima:** Writing – review & editing. **Toshihiko Nishioki:** Writing – review & editing. **Junko Watanabe:** Writing – review & editing. **Toshifumi Yae:** Writing – review & editing. **Takamitsu Banno:** Writing – review & editing. **Kenji Kido:** Writing – review & editing. **Kazuhisa Takahashi:** Writing – review & editing, Supervision.

## Funding statement

No funding was received for this study.

## Declaration of competing interest

The authors declare that they have no known competing financial interests or personal relationships that could have appeared to influence the work reported in this paper.
